# Body Water Distribution and Risk of Cardiovascular Morbidity and Mortality in a Healthy Population: A Prospective Cohort Study 

**DOI:** 10.1371/journal.pone.0087466

**Published:** 2014-02-03

**Authors:** Nikoline Nygård Knudsen, Thora Majlund Kjærulff, Leigh Cordwin Ward, Ditte Sæbye, Claus Holst, Berit Lilienthal Heitmann

**Affiliations:** 1 Institute of Preventive Medicine, Research Unit for Dietary Studies, Bispebjerg and Frederiksberg Hospitals as part of Copenhagen University Hospital, The Capital Region, Copenhagen, Denmark; 2 School of Chemistry and Molecular Biosciences, The University of Queensland, Brisbane, Australia; 3 The Boden Institute of Obesity, Nutrition, Exercise and Eating Disorders, Sydney University, Sydney, Australia; 4 National Institute of Public Health, University of Southern Denmark, Copenhagen, Denmark; 5 Research Centre for Prevention and Health, Copenhagen University Hospital, Glostrup, Denmark; Innsbruck Medical University, Austria

## Abstract

**Background:**

Early alterations in the cardiovascular structure and function may change normal body water distribution. The resulting fluid shifts may thus serve as an early marker for cardiovascular disease. However, studies examining this in healthy populations are absent.

**Objective:**

This study examined the association between the proportion of total body water that is extracellular water and subsequent development of non-fatal or fatal cardiovascular disease in a healthy population.

**Method:**

Bioelectrical impedance spectroscopy is an easy-to-use, non-invasive and relatively inexpensive technique to evaluate changes in body water distribution. A random subset (n = 2120) of Danes aged 41-71 years, examined in 1993–1994 for body water distribution by bioelectrical impedance spectroscopy was included. Cox-proportional hazard models and linear splines were performed. The ratio between resistance estimates from an infinite-frequency and from no-frequency (R_∞_/R_0_) was used as a surrogate measure of ratio between extracellular water and total body water. The outcome was 13.5 years of follow-up for cardiovascular morbidity and mortality.

**Results:**

A high proportion of total body water that is extracellular water was associated with increased risk of incident cardiovascular disease. A threshold effect was evident, with greatly increased risk of cardiovascular morbidity and mortality above R_∞_/R_0_ = 0.68. Below the threshold there seemed to be no additional benefit of having a low ratio.

**Conclusion:**

Our findings suggest that non-clinically evident oedema, measured as an increased proportion of total body water that is extracellular, above a threshold of 0.68, may be an early marker of pre-clinical cardiovascular disease. This simple, safe, cheap and easily obtainable measure of R_∞_/R_0_ from bioelectrical impedance may help the early identification of these otherwise clinically healthy individuals who are at an increased risk of future cardiovascular disease. However, more studies are needed before it can be concluded that bioelectrical impedance spectroscopy improves clinical risk prediction.

## Introduction

As body water distribution may change already in the very early phases during disease development, the possibility of measuring such early fluid shifts may be an attractive marker of preclinical disease. A number of studies have investigated the association between various measures of water distribution and health outcomes in different patient groups. For example, studies investigating fluid imbalances in patients with advanced colorectal cancer, lung cancer, breast cancer or pancreatic cancer have found lower survival among those patients with a high proportion of extracellular-to-intracellular water (ECW/ICW) determined by phase angle – an indicator of water distribution obtained from impedance technology [1]–[4]. The same association between fluid imbalance and prognostic outcomes (e.g. survival time) was found in studies investigating patients with conditions such as chronic obstructive pulmonary disease, acute heart failure, hepatitis C infection, HIV, renal diseases and patients receiving peritoneal dialysis [5]–[10]. Similarly, two studies investigating dengue virus infection in children or patients with suspected bacteraemia found an expansion of extracellular water (ECW) to be correlated with subsequent increased disease severity and mortality. These studies all found an enlarged ECW proportion to be associated with later disease severity and premature death in a variety of sick populations [11], [12]. Studies have also found body water balance and distribution to be associated with blood viscosity and the functioning of large arteries and thereby related to future risk of cardiovascular disease (CVD) [13], [14]. However, it remains to be investigated as to whether commonly detected alterations or differences in body water distribution in a *healthy population* may have future clinical implications.

We hypothesise that an increased ECW volume, expressed as an elevated extracellular to total body water ratio, may predict a dysfunctional cardiovascular system and hence *pre*clinical CVD. The aim of the present study was therefore to examine the association between water distribution, determined by bioelectrical impedance spectroscopy (BIS), and later morbidity and mortality from CVD in a random population sample of healthy individuals after accounting for covariates from a range of anthropometric variables and lifestyle factors.

## Materials and Methods

### Study population

The data was part of the Danish MONICA (Monitoring Trends in Cardiovascular Disease) study, which is a longitudinal population-based study sampled from the greater Copenhagen area. The first MONICA survey was carried out in 1982–1983 as an age-stratified sample of subjects born in 1922, 1932, 1942 and 1952 selected at random from the Danish Civil Registration System (n = 4807) [15]. Subjects not born in Denmark were excluded from the study (n = 226). Out of the 4581 subjects, 3608 (79%) underwent a general health examination. The present study was based on the data collection from the third survey conducted between May 1993 and November 1994 (n = 2656) and included measures of BIS [16]. All subjects with formerly registered cancer diagnoses or self-reported diabetes or intake of diuretics (n = 247), as well as subjects with a registered CVD diagnose before 1993–1994 (n = 190), were excluded from further study. Furthermore, only those with complete information on impedance measurements were included. The final sample included 822 women and 861 men, who were followed for development of incident CVD morbidity and mortality from examination until 8 July 2007.

### Endpoint

Information on non-fatal and fatal CVD during follow-up was retrieved from the Danish National Patient Register [17] and the Danish Register of Causes of Death [18]. Subjects were followed for an average of 13.5 years. For incidence of CVD, ICD-8 codes 390–458 and ICD-10 codes I00-I99 were used. Generally, there is minimal loss to follow-up from these registers because all Danish citizens have a unique personal identification number that renders linkage at the individual level between these nationwide registers and other data sources possible [19].

### Ethics Statement

The data collection was done with permission from the Danish Data Protection Agency. All subjects gave written informed consent and the project was approved by the Committee on Biomedical Research Ethics for the Copenhagen County. The project is in accordance with the Helsinki Declaration of 1975 as revised in 1983 on human rights.

### Bioelectrical impedance measurements

BIS is a convenient, safe and reliable method to assess total body water (TBW), extracellular water (ECW) and intracellular water (ICW) in a variety of clinical populations [16], [20]–[25].

Measurements of whole body, wrist to ankle, impedance were made using an SEAC SFB3 impedance spectrometer (SEAC-ImpediMed Ltd, Brisbane) and the tetrapolar method of electrode arrangement. The protocol for measuring BIS in this study was similar to that recommended by the European Society for Parenteral and Enteral Nutrition (ESPEN) for clinical applications [25]. Prior to examination, the subjects had fasted for 12 hours over night. The subjects were lying relaxed on a couch while attention was paid to the abduction of limbs, as, for reasons of conductivity, it is important that the legs are separated and that the arms are at a distance from the trunk. The same nurse took all measurements. Additionally, contact with any metal parts on the couch was avoided to eliminate electrical conduction. The environment was electromagnetically neutral with no strong electrical or magnetic fields and at an ambient temperature of approximately 23–24°C. Silver-silver chloride EKG-style gel electrodes were placed on the individual patient’s skin with two electrodes, through which the alternating electric current entered the body, attached to the dorsal surface of the right hand and foot, at the distal metacarpals and metatarsals, respectively. Two detection electrodes were placed on the wrist and the ankle, between the distal prominences of the radius and ulna at the wrist and the medial and lateral malleoli at the ankle. Resistance and reactance were measured at 496 discrete frequencies in the range 4–1012 kHz. From these data the resistance at zero (R_0_) and infinite frequencies (R_∞_) were determined according to Cole theory [26]. R_0_ can be used to predict ECW because cell membranes are impermeable to the current at very low frequency, while resistance at an infinite frequency (R_∞_) can be used to predict TBW because the cell membrane is permeable to the current at high frequency and hence flows through TBW [27]. Coefficients of variation of measurement have been determined previously [28]: 0.26% and 0.17% for R_0_ and R_∞_, respectively. In the present study the impedance ratio of R_∞_/R_0_, was used as a surrogate measure of the ratio ECW/TBW, henceforth referred to as the ECW proportion (note the inverse relation between ECW and R_0_, and TBW and R_∞_, respectively) [24].

### Covariates

The covariates were chosen a priori. Systolic and diastolic blood pressures were measured with a London School of Hygiene sphygmomanometer, with one of three different cuffs. Duplicate measurements were performed on the left arm after a minimum of five minutes’ rest in a supine position. Means of duplicate measurements were calculated. High-density lipoprotein (HDL), low-density lipoprotein (LDL), and triglyceride concentrations were measured from blood samples drawn after a 12-hour overnight fast. Commercial enzymatic methods (Boehringer Mannheim, GmbH, Mannheim, Germany) were used to analyse for the lipids. All anthropometric measures were taken at baseline in accordance with WHO standards [29].

Information on lifestyle factors was retrieved through a detailed self-administrated questionnaire at baseline. Information on smoking status from 1993–1994 and 1982–83 was used to calculate pack years of smoking (years of smoking x daily tobacco consumption (g) divided by 20 (cigarrettes per package)) for current and previous smokers. Alcohol consumption was included in the analysis as a continuous variable, where one unit of alcohol corresponds to 1 beer, 1 glass of wine or 4 cl. of spirits. Physical activity was categorised into sedentary (sedentary), light exercise (light activity at least 4 hrs/week), and hard exercise (strenuous exercise at least 3 hrs/week). Educational level was included in the analyses as a continuous covariate on a scale counting years of education. Finally, the questionnaire asked, “Has your doctor ever told you that you have had either: diabetes, coronary thrombosis, other heart disease, stroke, pulmonary thrombosis or embolus?”, with the response categories yes/no. Together with the health information from the Danish registers on previous diagnoses this information was used in order to exclude anyone with a known diagnosis from the study, so that only healthy individuals were followed up.

The information obtained from registers on previous diseases included all previous cancers from the Danish Cancer Registry [30], and all CVD from the Danish National Patient Register [17]. For incidence of cancer, ICD-7 codes 140–148, 150–165, 170–181 and 192–207 were used; for incidence of CVD, ICD-8 codes 390–458 and ICD-10 codes I00-I99 were used.

### Statistical analyses

We used Mann-Whitney U tests and χ^2^ tests to compare the distribution of the variables between those who experienced or died of CVD and the remaining subjects. Cox proportional hazard regression models were performed with age during follow-up as the underlying time scale [31]. Participants’ age at measurement of BIS defined the age at entry.

We fitted a series of four models examining the association between water distribution and CVD morbidity and mortality under different adjustments schemes. The crude Model 1 was adjusted for birth cohort and sex. Model 2 was adjusted for sex, birth cohort, alcohol intake, smoking, educational level, physical activity and BMI. Model 3 was additionally adjusted for systolic and diastolic blood pressure. Finally, Model 4 also included waist circumference and concentration of HDL, LDL and triglyceride. In order to examine potential sex differences in the association we also conducted the analyses for men and women separately. Furthermore, we conducted a sensitivity analysis with only fatal CVD cases as the outcome as well as two analyses that included information on measured hypertension at baseline: one analysis included hypertension at baseline as a confounder in the four models and the other analysis excluded subjects with hypertension at baseline.

Restricted cubic splines with three knots placed at impedance ratios of 0.65 (10^th^), 0.69 (50^th^) and 0.73 (90^th^) to allow for non-linear effects of the continuous effect variable were used to graph the hazard ratios (HR). The splines were restricted to being linear below the first knot and after the last knot. The curves had a shape indicating a threshold effect in the curve minimum. The minimum value was 0.6831 (hereafter 0.68) in the fully adjusted model with only slight deviation across the four models. In the final analyses HRs were calculated below and above the impedance ratio of 0.68. The proportional hazards assumption was examined with Schoenfeld residuals and found no indication of problems regarding non-proportionality [32]. Analyses were performed using STATA 9 and SPSS 17.0.

## Results

Over approximately 13.5 years of follow-up, 299 men and 227 women experienced or died of incident CVD. Table 1 provides information on the anthropometric variables and lifestyle factors for subjects with and without incident non-fatal or fatal CVD shown for men and women. Compared with the men who experienced or died from CVD during follow-up, baseline R_∞_/R_0_, BMI, waist circumference, systolic blood pressure, diastolic blood pressure, triglycerides and the amount of smoked pack years were generally lower for men who did not experience CVD, whereas baseline HDL, years of education and hard physical activity were greater for those who did not experience CVD. There was no significant difference in LDL or alcohol intake between the men who did not experience CVD and those who experienced or died of CVD. Similar observations were generally made for women, although not significantly so for BMI, HDL, pack years and physical activity. Among the women, those who experienced or died from CVD had a significantly higher baseline level of LDL compared to the women who did not experience CVD.

**Table 1:** Descriptive information of anthropometric variables and lifestyle factors (1993–1994) by sex and vital status (2007) in healthy Danes.

Variablesa, b		Men			Women		
		Censored	Sick or dead of CVD	P value^c^	Censored	Sick or dead of CVD	P value^c^
R_∞_/Ro		0.67 (0.62 to 0.72)	0.68 (0.63 to 0.74)	<0.001	0.70 (0.66 to 0.75)	0.71 (0.66 to 0.77)	<0.001
BMI (kg/m^2^)		25.9 (21.2 to 32.1)	26.4 (21.4 to 33.2)	0.01	24.0 (19.8 to 33.8)	24.7 (19.4 to 33.6)	0.23
Waist circumference (cm)		92.0 (77.0 to 111.0)	95.0 (80.0 to 114.0)	<0.001	78.0 (66.0 to 102.0)	81.0 (67.0 to 104.0)	0.003
Systolic blood pressure (mm Hg)		125 (108 to 155)	136 (112 to 175)	<0.001	123 (102 to 155)	135 (107 to 173)	<0.001
Diastolic blood pressure (mm Hg)		80 (67 to 97)	85 (69 to 103)	<0.001	78 (63 to 95)	81 (64 to 98)	0.001
HDL cholesterol (mmol/l)		128 (89 to 198)	123 (81 to 187)	0.02	155 (104 to 235)	153 (98 to 239)	0.16
LDL cholesterol (mmol/l)		399 (261 to 577)	409 (266 to 594)	0.11	376 (230 to 569)	404 (262 to 576)	<0.001
Triglycerides (mmol/l)		125 (61 to 320)	136 (69 to 359)	0.003	102 (55 to 227)	122 (65 to 284)	<0.001
Years of education		10.0 (7.0 to 19.0)	9.0 (7.0 to 17.0)	<0.001	10.0 (7.0 to 17.0)	9.0 (7.0 to 16.0)	0.006
Smoking (pack years)		13.9 (0.0 to 50.0)	22.9 (0.0 to 69.0)	<0.001	4.2 (0.0 to 42.0)	6.3 (0.0 to 42.0)	0.31
Alcohol (units/week)		10.0 (0.0 to 39.0)	10.5 (0.0 to 50.0)	0.69	4.0 (0.0 to 20.0)	4.0 (0.0 to 21.0)	0.40
No (%) in each cohort	Born 1922	46 (8.2)	79 (26.4)	<0.001	54 (9.1)	70 (30.8)	<0.001
	Born 1932	119 (21.2)	77 (25.8)		126 (21.2)	50 (22.0)	
	Born 1942	185 (32.9)	87 (29.1)		185 (31.1)	55 (24.2)	
	Born 1952	212 (37.7)	56 (18.7)		230 (38.7)	52 (22.9)	
No (%) in level of physical activity	Sedentary	111 (20.0)	55 (18.7)	0.004	134 (22.8)	62 (27.9)	0.12
	Light exercise	264 (47.5)	172 (58.5)		354 (60.2)	133 (59.9)	
	Hard exercise	181 (32.6)	67 (22.8)		100 (17.0)	27 (12.2)	

a Figures are medians (5^th^ and 95^th^ centiles) unless stated otherwise.

b Number of subjects with information on each variable range from 1656 to 1683.

c Significance assessed using Mann-Whitney U tests and χ^2^ tests.

**Figure 1:**
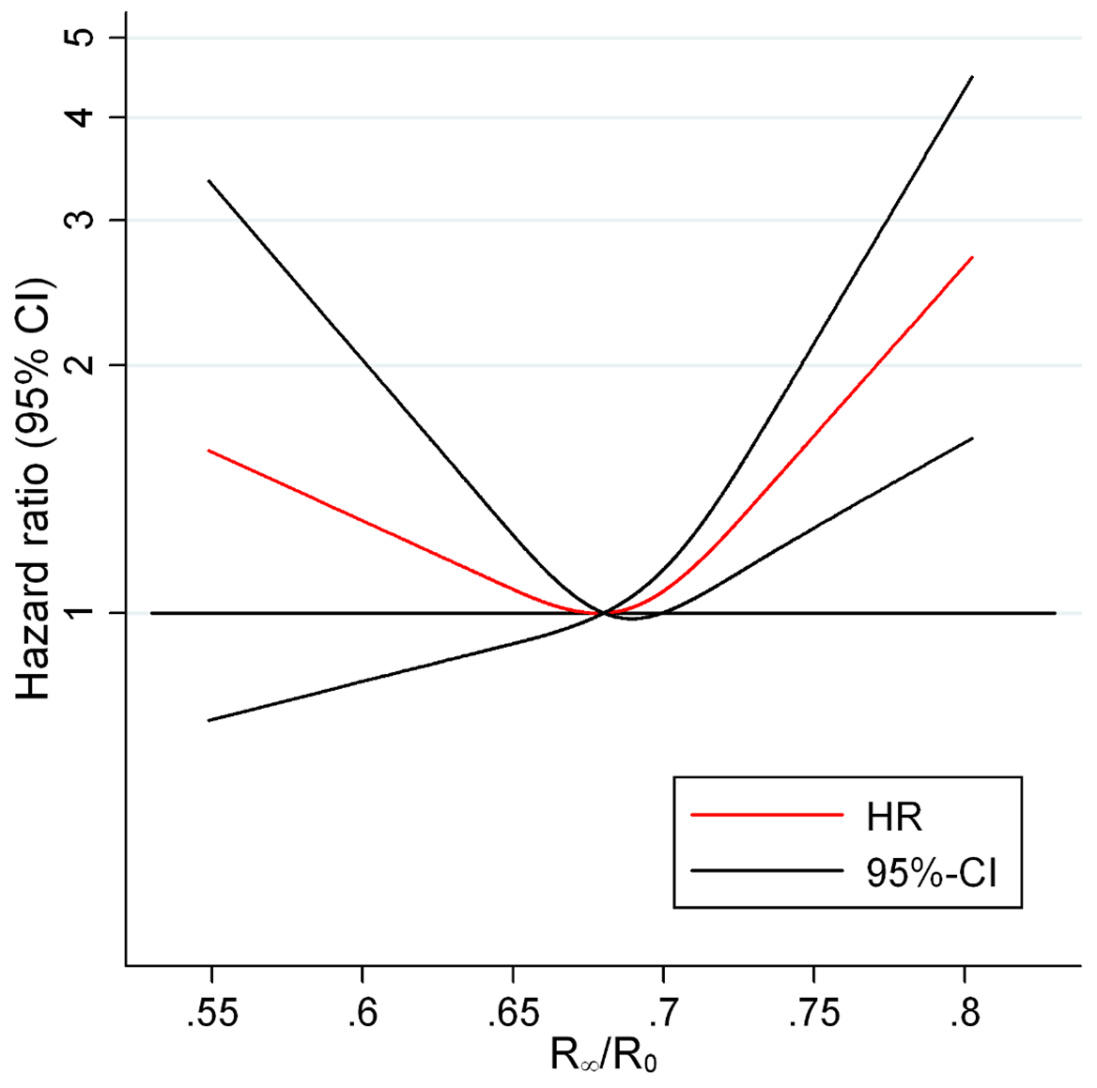
HRs for CVD morbidity and mortality according to impedance ratio in a semi-adjusted model. Figure 1 illustrates the HRs for CVD morbidity and mortality as a function of impedance ratio among 1683 healthy subjects when adjusting for birth cohort and sex. The curve shows that a threshold effect for high impedance ratio on CVD morbidity and mortality was evident at around 0.68; below this threshold the risk of having a higher ECW proportion carried no further risk of CVD morbidity or mortality. Abbreviations: CVD (cardiovascular disease), R_∞_/R_0_ (impedance ratio).

**Figure 2:**
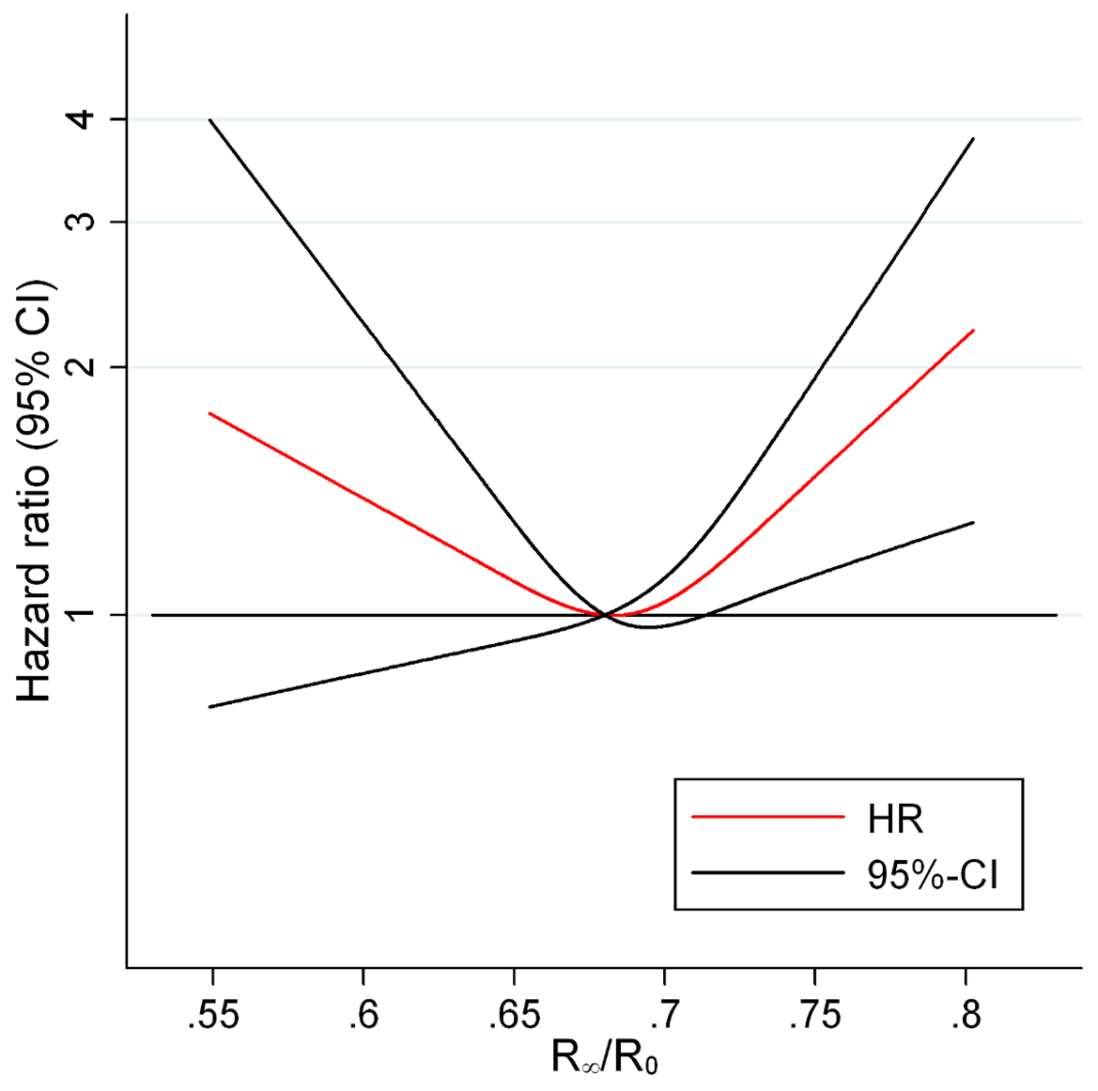
HRs for CVD morbidity and mortality according to impedance ratio in a fully adjusted model. Figure 2 shows the HRs for CVD morbidity or mortality according to impedance ratio among 1622 healthy subjects when adjusting for birth cohort, sex, alcohol, smoking, educational level, physical activity, BMI, systolic and diastolic blood pressure, HDL, LDL, triglycerides and waist circumference. The figure illustrates that the association between the impedance ratio and CVC morbidity and mortality remains after adjusting for potential confounders. Abbreviations: CVD (cardiovascular disease), R_∞_/R_0_ (impedance ratio).

The association between ECW proportion and CVD morbidity and mortality was examined in four different adjustment models. Figure 1 shows the predicted HRs and confidence intervals for having a high proportion of ECW on subsequent development of non-fatal or fatal CVD for the crude Model 1. Above an impedance ratio, R_∞_/R_0_, of 0.68, the risk of morbidity and mortality from CVD was significant and increased steadily, whereas below this value there was no significant association between the impedance ratio and risk of morbidity and mortality from CVD indicating a threshold effect at 0.68. The shape of the curves was essentially similar before and after adjustment for covariates as seen in Figures 1 and 2 (fully adjusted Model 4). Only small differences in HRs for R_∞_/R_0_ above 0.68 could be observed for the four different models.

Tables 2 shows the predicted HRs and confidence intervals for various centiles in the distribution of the impedance ratio (the 2.5^th^, 5^th^, 10^th^, 25^th^, 50^th^, 75^th^, 90^th^, 95^th^ and 97.5^th^ centiles) in relation to cardiovascular morbidity and mortality. The reference HR is for those with a median impedance ratio, which in this case is close to the threshold value. An impedance ratio above median was related to an increased risk of cardiovascular morbidity and mortality. The quarter of subjects with the highest impedance ratio had more than a 10% increased risk of CVD morbidity and mortality compared to those with a median impedance ratio. Furthermore, the 10% of subjects with the highest impedance ratio had around a 30% increased risk of CVD morbidity and mortality and the 5% of subjects with the highest impedance ratio had an increased risk of approximately 50%.

**Table 2:** Figures are HRs (95% confidence intervals) for associations between impedance ratio (in centiles) and cardiovascular morbidity and mortality among healthy Danes 1993/94–2007.

Centiles (impedance ratio)	Model 1^a^	Model 2^b^	Model 3^c^	Model 4^d^
2.5 (0.624)	1.16 (0.83 to 1.63)	1.22 (0.86 to 1.73)	1.25 (0.88 to 1.76)	1.24 (0.86 to 1.79)
5 (0.634)	1.12 (0.85 to 1.47)	1.16 (0.88 to 1.54)	1.18 (0.89 to 1.57)	1.18 (0.87 to 1.59)
10 (0.645)	1.07 (0.87 to 1.32)	1.11 (0.89 to 1.37)	1.12 (0.90 to 1.39)	1.12 (0.89 to 1.41)
25 (0.666)	1.00 (0.91 to 1.10)	1.01 (0.92 to 1.12)	1.02 (0.93 to 1.13)	1.03 (0.92 to 1.14)
50 (0.690)	1	1	1	1
75 (0.712)	1.14 (1.06 to 1.23)	1.14 (1.05 to 1.23)	1.12 (1.03 to 1.21)	1.10 (1.02 to 1.20)
90 (0.733)	1.37 (1.17 to 1.62)	1.37 (1.16 to 1.63)	1.32 (1.11 to 1.56)	1.28 (1.08 to 1.53)
95 (0.746)	1.56 (1.24 to 1.95)	1.56 (1.23 to 1.97)	1.48 (1.17 to 1.87)	1.42 (1.12 to 1.81)
97.5 (0.760)	1.78 (1.33 to 2.39)	1.79 (1.32 to 2.43)	1.67 (1.23 to 2.27)	1.59 (1.16 to 2.17)

a Adjusted for birth cohort and sex.

b Additionally to *a* adjusted for alcohol intake, smoking, educational level, physical activity and BMI.

c Additionally to *b* adjusted for systolic and diastolic blood pressure.

d Additionally to *c* adjusted for waist circumference, level of HDL and LDL and concentration of triglycerides.

In order to examine whether the effect of a high impedance ratio on CVD morbidity and mortality differed by sex, the analyses were conducted for men and women separately; however, associations were essentially similar for both sexes. Moreover, the interaction between the impedance ratio and sex was included in all models. Neither the estimated HRs of the interaction nor the graphical display of the curves for men and women provided evidence for sex differences in the observed association. Finally, sensitivity analyses were made for the fatal CVD endpoints exclusively. In total only 52 men and 30 women died from CVD during follow-up. These additional analyses generally showed no significantly increased hazard among the subjects in the groups with the highest centiles of the impedance ratio.

More than 1 out of 6 subjects in the study population had moderate hypertension (>140/90) when examined in 1993–1994. However, results were similar after further adjusting for presence of hypertension. Additionally, an analysis excluding individuals with hypertension gave approximately the same results.

**Figure 3:**
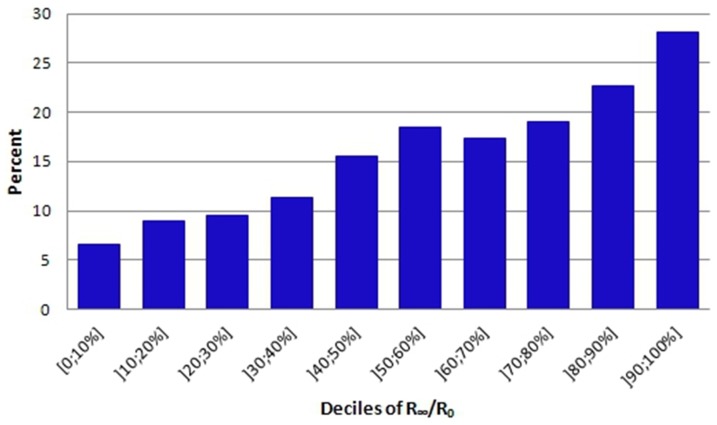
Deciles of R_∞_/R_0_ in relation to self-reported swelling of the legs during the day (%). Figure 3 shows the percentage of the study population that reports swelling of the legs during the day in each of the impedance ratio deciles. The figure illustrates a rise in the incidence of swollen legs with increasing impedance ratio. Abbreviations: R_∞_/R_0_ (impedance ratio).

Oedema is a clinical indicator of malfunction in the mechanisms regulating body water distribution. In the self-reported questionnaire the subjects responded to the question, ”Do your legs swell during the day?” with the response categories yes/no. A total of 264 subjects, corresponding to 15.7% of the study population with information on this question (n = 1677), indicated that they had swollen legs. When examining the association between swollen legs and the impedance ratio we found that the incidence of swollen legs rose with increasing impedance ratio. However, a clinical focus on swollen legs is not sufficient to identify all subjects who, according to our model, are at high risk of CVD morbidity and mortality seeing that among subjects in the highest decile of impedance ratio only around 28% reported swollen legs (Figure 3).

We performed a non-response analysis using Mann-Whitney U and χ^2^ comparing those excluded due to incomplete impedance measurements with those having complete information on all impedance measures. We found no significant differences in the distribution of covariates between the groups except that subjects with complete impedance measures included in this study had significantly higher systolic and diastolic blood pressure and tended to be slightly younger.

Furthermore, the number of subjects who experienced or died of CVD was not significantly different when comparing those with and without complete impedance measurements.

## Discussion

In a population sample of healthy adult Danes, we demonstrated a strong and independent association between excess ECW, as measured by the impedance ratio, and development of non-fatal or fatal CVD over the subsequent almost 13.5 years, suggesting that early sub-clinical fluctuations in water balance detectable with BIS may be used to identify otherwise healthy subjects who are at high risk of subsequently developing CVD. The association was evident when R_∞_/R_0_ was above a threshold value of 0.68, indicating the existence of a critical ECW proportion for increasing the risk of CVD morbidity and mortality. Below this threshold we did not find the impedance ratio to be significantly associated with CVD morbidity and mortality. Almost half of the population had an impedance ratio above the threshold, suggesting that the measure may have great clinical relevance.

The observed association remained remarkably stable after controlling for competing risk factors of CVD, including hypertension, and a range of socioeconomic factors, thus suggesting that early fluid disturbances leading to excess ECW may serve as a biomarker for future CVD. In fact the observed fluid distribution shifts may to some extent specifically be an easily obtainable marker of early stages of, and yet undiagnosed, chronic heart failure, that may otherwise only have been discovered by a screening of this healthy population subset, using one or more of the novel biomarkers like N-terminal pro-brain natriuretic peptide [33]. Future studies may use measures like N-terminal pro-brain natriuretic peptide to verify whether or to what extent BIS may be used to assess specifically undiagnosed chronic heart failure.

Moreover, the analyses did not show any sex difference in the association between body water distribution and CVD morbidity and mortality, thus indicating no difference in threshold values for men and women.

### Pathophysiological mechanisms

Earlier studies have suggested that the ratio between ECW and TBW could be used as a prognostic indicator when illness is *already* present. This study opens new potentials in relation to prevention of CVD among *healthy* individuals as the ECW proportion was found to be an early marker of undetected disease. The results of the present study do not answer whether a change in water distribution *per se* increases the risk of CVD or whether the expanded ratio points toward an underlying disease process. If the first suggestion is the case, it could enable a reduction in the risk of CVD by lowering extracellular fluid overload. If the second proposal is the case, the BIS method may be used to identify preclinical disease in apparently healthy subjects before CVD becomes manifest.

The pathophysiological mechanisms linking fluid balance to CVD are not fully understood. One plausible causal pathway relates extracellular fluid overload to hypertension and left ventricular hypertrophy, which are both traditional risk factors of CVD [34]. However, our results were unaffected by the presence of hypertension. Equally, changes in fluid distribution have consequences for electrolyte and acid-base balance [35]. However, future studies are needed to clarify the pathophysiological mechanisms and further investigate the threshold effect, as well as the association between the impedance ratio and more specific CVD outcomes.

### The applicability of BIS and the interpretation of the impedance ratio

Many studies have found good agreement of water volume predictions obtained from spectroscopy measures by BIS and tracer dilutometry at the population level [23]. Along with a range of practical advantages these findings make BIS a preferable method for determining water distribution in larger population samples.

The concept of using the ratio of impedances measured at high and low frequencies as a surrogate marker for the ratio ECW/TBW is not new [24], [36]. In the present study we used R_∞_ and R_0_, the theoretically optimal measures of TBW and ECW, respectively [27]. Alternatively, an advantage of utilising population-specific regression equations to convert the measured impedances to absolute volumes of ECW and TBW, is that the equations provide measurements that are familiar and intuitively easy to comprehend and communicate to non-specialists of BIS. Evaluating water distribution using raw impedance measurements, instead of computed volumes derived from regression models obtained in restricted samples of human subjects, however, avoids the possibility of inadvertently using an inappropriate prediction algorithm.

Interpreting the impedance ratio should be done with caution as it represents the proportion of ECW to TBW, rather than an index of volume status. An increase in the impedance ratio might result from an expanded volume of ECW or a decreased proportion of TBW due to a reduction of ICW. Indeed, an increased impedance ratio may reflect hypervolemia, or it can indicate malnutrition characterised by changes in cellular membrane integrity and alterations in the fluid balance thereby decreasing the body cell mass [37], [38].

### Study strengths and limitations

A major strength of the present study is the prospective design and the large size of the population with complete impedance measures, which provided a unique opportunity to investigate our hypothesis. The non-response analysis showed no substantial differences when comparing those excluded due to incomplete impedance measures with the study population, thus indicating that selection bias did not seem to be an issue in the present study.

It may be argued though, that the exclusion of subjects with disease was incomplete as it was based on a restricted number of diagnoses. With this procedure a limited number of sick subjects, e.g. chronic haemodialysis patients, who have been found to have a higher ECW proportion compared to healthy subjects [39], could have been incorrectly categorised as healthy and thus included in the study population. However, the exclusion of subjects who reported intake of diuretics probably eliminated most of the chronic haemodialysis patients who furthermore represented only 2039 patients in Denmark (equivalent to 0.037% of the total Danish population (5.45 million)) in 2007 [40]. Inclusion of subjects undergoing haemodialysis was therefore not likely to have confounded our results substantially. Also, all subjects with any previous CVD diagnosis for which they were hospitalized, were excluded, thus eliminating the possibility that any subject with a known diagnosis remained for follow up and subsequent analyses.

## Conclusion

The present study found that among a healthy subset of adult Danes a high proportion of ECW was associated with the development of cardiovascular morbidity and mortality. The 10% of subjects with the highest impedance ratio had an approximately 30% increased risk of developing non-fatal or fatal CVD over the following 13.5 years. The increased risk was independent of lifestyle and cardiovascular risk factors such as obesity, blood pressure and cholesterol. Furthermore, a threshold effect was evident, which suggests that the association was substantial for water distribution ratios above 0.68. This study adds to the growing body of evidence regarding the clinical application of BIS-derived measures of water distribution. If future research can confirm the hypothesis of the present study, which suggests an increased R_∞_/R_0_ to be a cheap, easily obtainable and safe marker of preclinical CVD, the results may help general practitioners to identify otherwise healthy individuals with an increased risk of future CVD and early mortality.
